# Influences of Built Environment with Hilly Terrain on Physical Activity in Dalian, China: An Analysis of Mediation by Perceptions and Moderation by Social Environment

**DOI:** 10.3390/ijerph16244900

**Published:** 2019-12-04

**Authors:** Peijin Sun, Wei Lu, Yan Song, Zongchao Gu

**Affiliations:** 1 Research Section of Environment Design, School of Architecture and Fine Art, Dalian University of Technology, Dalian 116023, China; peijinsun@mail.dlut.edu.cn (P.S.); gzc1001@dlut.edu.cn (Z.G.); 2 Department of City and Regional Planning, University of North Carolina at Chapel Hill, Chapel Hill, NC 27514, USA; ys@email.unc.edu

**Keywords:** built environment, physical activity, perceived environment, social environment, sense of community, BMI, mediation effect, geographic information systems

## Abstract

Neighborhood built environment may influence residents’ physical activity, but evidence of non-major Chinese cities is lacking. We investigated the impact of five socio-demographic characteristics, 10 objectively assessed environment characteristics, eight perceived neighborhood attributes, and social environment on physical activity and health outcomes (sense of community, body mass index, as well as self-reported health status). We also examined (1) five conceptually comparable perceived neighborhood attributes as mediators of the relationship between objective environment attributes and physical activity; (2) other perceived indicators and social environment as moderators of those relationships, using the mediation analysis in regression. Objectively assessed residential density, land use mix, street connectivity, and accessibility were curvilinearly and/or linearly related to physical activity. The slope of terrain was inversely associated with body mass index (BMI). None of the perceived attributes were found as mediators probably due to the weak associations between subjective and objective environments. High density facilitated physical activity but hindered the sense of community. Further, the perceived aesthetic and safety were associated with physical activity. Additionally, social environment moderated the positive associations of all perceived environments (except for slope) and sense of community. The present study demonstrated that both physical and social environment attributes significantly correlated with physical activity in Dalian.

## 1. Introduction

Physical inactivity can be a fundamental cause of various public health problems, especially chronic diseases. In countries with fast-growing economies, the decline in physical activity is even more pronounced. For example, the level of physical activity of 1.3 billion people in mainland China is experiencing a steeper decline than in any other country: the level of physical activity has dropped by 45% in less than a generation (18 years) [1]. Numerous studies have shown that regular physical activity can effectively reduce the risk of preventable chronic diseases [2,3,4,5]. Environmental improvements can provide health benefits by promoting physical activity. However, the urban form and social environment of Chinese cities are significantly different from that of many Western cities. It is not clear whether the associations between environmental characteristics and physical activity from Western cities can be directly applied to China [6]. For example, previous studies on transport-related physical activity in China have shown little association with built environment [7], because the most common form of it is the daily commute to workplace [8]. Besides, density might also impede walking and other outdoor sports activities [9,10], which is not in line with the existing literature. Extremely high density limits open spaces and parks, which could lead to serious traffic barriers, safety, and pollution problems [6,11]. However, evidence of non-major Chinese cities, with lower density than first tier cities, is lacking. Therefore, this study provides some insights into the interaction among various factors associated with physical activity and health outcomes in Chinese non-first-tier cities.

Evidence has reported the association between built environment and physical activity, with land use mix, density, and connectivity as pivotal determinants of physical activity [12,13]. Other walkability related factors, such as parks and squares, and pedestrian infrastructure also affect physical activity [14]. Besides, easy access to parks and other recreation facilities has been associated with more leisure-time physical activity [15]. Adults tend to be more physically active when they live in higher density, mixed-use neighborhoods with destinations such as shops and parks within walking distance. However, findings have been inconsistent, perhaps owing to multiple modes of assessment and overreliance on self-reported measures [13].

On the other hand, objective environment attributes tend to affect physical activity through people’s perception of the environment according to ecological models of health behavior. The results of objectively-assessed and perceptions of the same built environment do not have high consistency in physical activity [16], as perception may be influenced by personal cognitive and emotional factors [17]. Only 8.2% of the environmental factors are related to the same result. The influence of perceived environment on physical activity is slightly higher than that of the conceptually comparable objectively-assessed environment [18]. However, few studies have demonstrated the mediation and moderation effect of neighborhood environment and physical activity relations. Research has documented that the association between the number of parks and moderate and vigorous physical activity was mediated by its perceived indicator [19], facility count and population density were associated with neighborhood walking and physical activity via the perceived neighborhood environment [20]. While, the association between built environment and physical activity varies according to the individual socioeconomic backgrounds, such as gender, age, and education, but there is no consistency [21,22,23]. Higher socioeconomic status may lead to higher physical activity [24]. In addition, the associations of objective intersection density and land use mix with moderate and vigorous physical activity are moderated by both gender and perceived pedestrian infrastructure/safety [19]. Plus, education and gender moderate the association between safety from crime and meeting high physical activity levels [21]. Clear relationships between perceived neighborhood features and built environment with physical activity levels remain elusive [19,25] 

In addition to built environment, individual (physical and psychological) and social environment have multilevel interactions with physical activity according to the ecological model [26]. Social determinants of health also have robust associations with health status, including a wide range of elements, such as socioeconomic status, social networks, social support, social cohesion, social capital, and so on [27]. Social capital is the most used which contributes to various health outcomes [28] and physical activity [29,30]. Previous studies examining the association of social environment on health outcomes emphasize different aspects of the social environment [31,32]. The social factors that can affect health are the degree, intensity, and quality of our social connection with others [33]. This paper focused on the connections between residents and neighbors. However, too little work has been devoted to the effect of social environment as a moderator between built environment and physical activity, especially in China. The interaction among various factors is still the least understood [21].

Given these considerations, this paper aims to explore whether objective measures of neighborhood built environment are (1) directly associated with, (2) indirectly associated with physical activity and health outcomes via the perceived environment, and (3) whether perceived environment is directly associated with physical activity and health outcomes, all controlling for demographic characteristics and taking social environment as the moderator. We conduct a series of mediation models to test the cross-level relationships between the different aspects of built environment and physical activity. The results provide an evidence base for community development in Dalian, China.

## 2. Materials and Methods 

### 2.1. Study Design and Participants 

**Figure 1:**
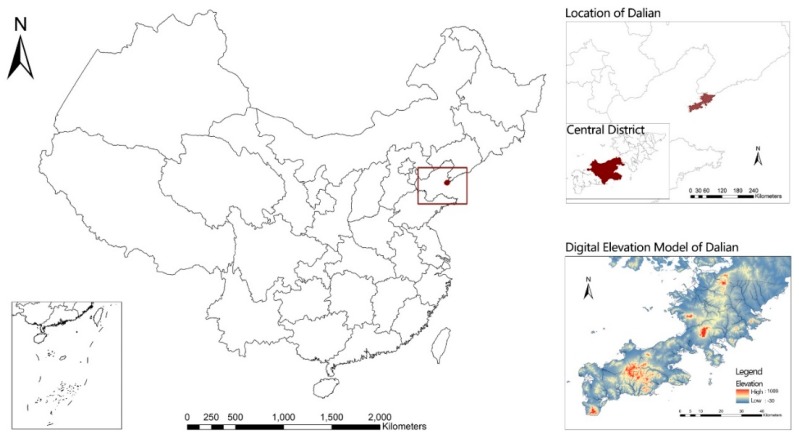
The location and digital elevation of Dalian.

**Figure 2:**
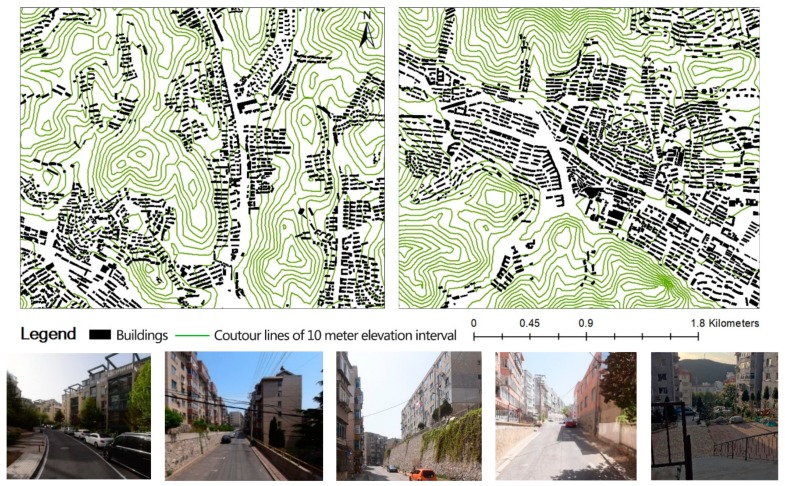
Examples of site plans of the community with slope or terraced space and photos (maps and images by authors).

We conducted the survey in Dalian, situated in the Northeast coastal area of China (Figure 1). Due to the hilly terrain, more than half of the residential communities have different forms of the slope (Figure 2), which may lead to obstacles in driving and walking. In particular, the average topographic elevation range in the study sample reached 40 m. The study sample was drawn from the four urban districts, including Zhongshan, Xigang, Shahekou, and Ganjingzi districts, which had approximately 2 million people and covered an area of 600 km^2^. The “neighborhood” is defined as an area within a 10–15 min walk from home. The study sample was restricted to individuals aged 22–64 that had lived in the neighborhood for at least one year. University students aged 18–22 were not eligible for this study because they tend to live on campus or study in other cities [8]. Participants also excluded the special age group, like people over 65, owing to the different recommended amounts of physical activity to stay healthy [34].

We developed an online survey that was completed by participants over a two-month time frame between July and August 2018. The survey was advertised through Weibo and Wechat (Chinese main social media platforms) public accounts. The online survey instrument consisted of four sections: general socio-demographic characteristics, individuals’ perceptions of their neighborhoods, participation in physical activities, and health outcomes. In total, 890 survey respondents started the online survey. Data cleaning was the key step to ensure the data quality of this paper, including the elimination of missing values, outliers, and error values. Only 649 target samples had a particular time of physical activity and eligible dwelling address (i.e., within the four urban districts and specific enough to be geocoded).

### 2.2. Measures 

#### 2.2.1. Outcome Assessment

The dependent variables included (1) frequency and duration of moderate and vigorous physical activity, (2) frequency and duration of walking, and (3) health outcomes. The modified version of the International Physical Activity Questionnaire (IPAQ) short version was used to measure total walking and physical activity during the past 7 days. Participants were first asked whether they had participated in at least 10 min of walking, moderate physical activity (MPA), or vigorous physical activity (VPA) over the past 7 days. Those who said yes to any of them continued with the questions. Questions included how many days they walk or do moderate/vigorous physical activities, and how much time they usually spent on one of those days doing activities. Response options ranged from 0 to ≥60 min/week. Further, we multiplied activity frequency values by the midpoint of the range of hours reported for a given activity (e.g., 30–45 min = 37.5 min) to calculate total physical activity time (min/week). 

Health outcomes contained both physical (body mass index, BMI) and mental aspects (Sense of Community, SoC). Body mass index was calculated as weight divided by the square of height (kg/m^2^). Sense of community can be an indicator of social capital [35]. Sense of community had a decisive influence on mental health [36] and was also associated with walking [37]. In this paper, sense of community was the product of a four-item Likert scale combining two items into one scale, including “Living in my neighborhood gives me a sense of community”, and “I would be willing to work together with others on something to improve the living environment in my neighborhood”. Additionally, participants’ previous chronic disease (i.e., mental disease, diabetes, or cardiovascular and cerebrovascular diseases) were included in the questionnaire.

#### 2.2.2. Socio-Demographic Characteristics 

Socio-demographic covariates were age (22–32; 33–42; 43–52; 53–64); gender (men; women); educational attainment (middle school; high school; junior college; university; master or higher); economic-level (low; medium-low; medium; medium-high; high); and car ownership (one or more; none). All socio-demographic characteristics were also considered as potential moderators. We controlled for demographic covariates that may confound associations between neighborhood environment, physical activity, and health outcomes. 

#### 2.2.3. Perceived Neighborhood Environment 

The abbreviated Chinese version of the Neighborhood Environmental Walkability Survey (NEWS-A) was used to assess an individual’s perceptions of his/her neighborhood that was determined to be valid and reliable in a Chinese population [38]. After translation, NEWS-A and the IPAQ were tested for their reliability and validity. Participants were asked to evaluate their neighborhood by responding to statements concerning various environmental attributes. The response format was a four-point scale ranging from “strongly disagree” (score 1) to “strongly agree” (score 4). Negative items were reverse-coded to match the remaining items, which included the following: traffic nearby streets, steep streets, major barriers, separated sidewalks by parked cars or grass/dirt strip. In this study, not all parts of the NEWS-A questionnaire items were selected. Particularly, items included those were conceptually comparable to the objective indicators (street connectivity; proximity to public transport stop; proximity of park/public facilities; slope) and other aspects (aesthetic; sidewalks; safety). Social environment factors were also added to the questionnaire, including social interaction (reflected by interactions with neighbors) and social activities (reflected by the richness and participation of activities in the community).

#### 2.2.4. Objective Built Environment

We measured 10 built environments, including land use mix, density (residential density and floor area ratio, FAR), connectivity (road network density and number of intersections), accessibility (number of public facilities and transit stops), slope, construction quality (housing price and built year), using geographic information systems (GIS) software. 

The land-use mix was represented by the “Frequency Density” of each land use unit. Land use mix is the most commonly used index. Land use mix degree can be calculated in three ways; accessibility, intensity, and form [39]. Frequency Density represented the quantity or density of specific destinations and the proportion of different land use within the study area. The land use form identification was conducted through BAIDU POI (an open database of Chinese mapping service), data describing facility locations. The POI data was divided into six types; residential (containing 19,677 data points), commercial and business facilities (containing 31,176 data points), green space and plaza (containing 303 data points), industrial (containing 11,255 data points), administration and public service (containing 15,067 data points), street and transportation (containing 5737 data points), depending on Chinese land use classification [40]. For each functional unit, the index Frequency Density and Category Ratio was constructed to identify functional properties. The calculation formula was as Equations (1) and (2):

(1) $$F_{i}=\frac{n_{i}}{N_{i}}\left(i=1,2,\ldots ,6\right),$$

(2) $$C_{i}=\frac{F_{i}}{\sum_{i=1}^{6}F_{i}}\times 100\%,\;i=1,2,\ldots ,6,$$

where *I* stands for POI type; *n_i_* indicates the number of POI of type *i* in the unit; *N_i_* means the total number of POI of type *i*; *F_i_* denotes the frequency density of POI of type *i* in the total number of POI of this type; *C_i_* refers to the proportion of the frequency density of type *i* in all types of POI in the unit.

Street connectivity was measured as the number of street intersections and road network density and the number of intersections, based on road network data from ESRI Street Map. Intersection count was defined as the number of three-way or greater intersections within the buffer. Facility count (number of public facilities within the buffer) was derived from the Baidu POI, which included data related to physical activity (e.g., parks, gyms, fitness places, or recreation centers). We assumed that these resources would be correlated with physical activity. 

Especially, we used the standard deviation of the elevation within the buffer area to represent the variations of the slope, with the data derived from Google Map. Additionally, the average housing price and construction year were used to reflect the general construction quality of the residential unit, derived from Lianjia (Real estate firm in mainland China). 

We created a 400-m radius buffer around each geocoded home address. Generally, most people prefer to walk within 400 m (a 5-min walk distance). Therefore, 400 m has been used as the value of acceptable walking distance, and features of buffers larger than 400 m have also been linked to walking [41]. An individual will walk up to 1600 m to reach a destination, but the most proper spatial context is unclear for understanding the relationship between the built environment and health [42]. Particularly, under 400 m scale, there were more built environment elements associated with the perceived environment and health results, compared with other scales (800, 1200, and 1600 m, or 10, 15, and 20 min walk distance) in author’s other research. Accordingly, we used 400 m as a radius to establish the buffer zone in this paper. 

Data were analyzed using ArcGIS 10.5 (Environmental Systems Research Institute, Redlands, CA, USA), and PROCESS© v3.1 (Andrew F. Hayes, NY, USA) for SPSS® v24.0(Armonk, NY, USA).

### 2.3. Statistical Analysis 

#### 2.3.1. Data

We adjusted the NEWS-A scale to better reliability and validity. First, Bartlett’s test of sphericity and Kaiser-Meyer-Olkin (KMO) factor analysis were performed on the data, with the results fitting for factor analysis (KMO = 0.814). Then, we used the Principal Component Analysis method to test validity. Accordingly, we eliminated the items which (a) by themselves created a component and (b) did not load on the un-rotated or initial component. The deleted item was dead-end streets. After adjustment, the overall reliability of the questionnaire items showed a value of 0.806, and five common factors were identified. However, the five components’ dimension was not completely consistent with the original dimension in NEWS-A scale (Appendix A). Next, we made the related items in each component into one scale using the scoring system for NEWS-A. Ultimately, perceived neighborhood attributes included accessibility and street connectivity (related to each other), walking obstacles (containing slope and obstacles), pedestrian environment, aesthetics, and safety. 

We also calculated Z-scores of all independent variables for data standardization to be used in regression models. Besides, logarithmic transformation of dependent variables (three types of the total time of physical activity) were carried out due to its skewed distribution.

#### 2.3.2. Analysis

The data analysis procedures were divided into three steps. First, one-way ANOVA was used to differentiate the health outcomes and perceived neighborhood environment among different social parties. Second, multiple logistic regression was used to examine the effects of different physical activity behaviors and health indicators on self-reported health outcomes. Third, we used a four-step mediation regression to analyze the effects of the built environment on physical activity and health outcomes.

This paper tested mediation with regression analysis in four steps. We first (Equations (3) and (4)) tested the correlations between objectively-assessed environment variables (independent variables, X) and physical activities (dependent variables, Y), as well as taking the corresponding perceived environment attributes as the mediator variable (mediators, M). This process can compare the relationship between the objective and perceived environment (whether the coefficient is significant) and examine the mediation effect (a or b_2_ are significant). We then (Equation (5)) examined mediation effects controlling for socio-demographic variables (covariables, Co) in step 2, if mediation effects were identified in step 1.

Y = Intercept + B(X) + e(3)

Y = Intercept_1_ + b_1_(X) + b_2_(M) + e_1_  M = aX + e_2_, (4)

Y = Intercept_2_ + b_3_(X) + b_4_(M) + b_5_(Co) + e_3_. (5)

We took (Equations (6) and (7)) the social environment variables as moderators (moderators, Mod) for the significant independent variables in step 3, if no mediation effects were identified. In the final step 4 of the analyses, we regressed physical activity (Y) onto the built environment variables (X) and conceptually comparable perceived environment attributes (M), social environment variables (Mod), and when appropriate, the significant socio-demographic variables examined previously as co-moderators (W).

Y = Intercept_3_ + b_6_(X) + b_7_(Mod) + e_4_, (6)

Y = Intercept_4_ + b_8_(X) + b_9_(Mod) + b_10_(X × Mod) + e_5_, (7)

Y = Intercept_5_ + b_11_(X) + b_12_(Mod/M) + b_13_(W) + b_14_(X × Mod/M) + b_15_(X × W) + e_6_. (8)

**Figure 3:**
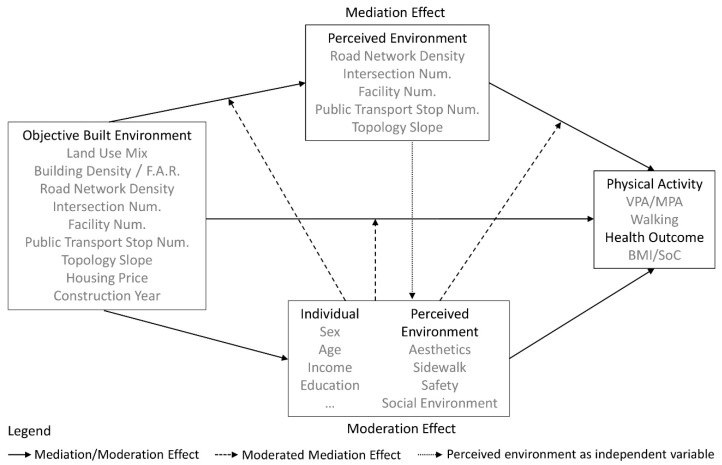
Conceptual framework of indirect and direct associations between objective built environment and health outcomes mediated/moderated by perceived environment. Note: Model depicts conceptually comparable perceived neighborhood environment as a potential mediator (Equations (1) and (2)), and socio-demographic, perceived environment, and social environment as moderators (Equations (3) and (4)) of the association between objective built environment and health outcomes. The dashed lines and arrows also show the moderated mediation effect (Equation (5)), that is, the potential moderators may influence the mediation effect.

All regression models were constructed in three groups (Figure 3). We first tested the mediation effect with the objectively assessed environment as the independent variable and physical activity as the dependent variable. We then took the perceived environment attributes as the independent variable. Next, we examined the health outcomes as the dependent variable. If an effect was not moderated or mediated, we removed the nonsignificant interaction term from the model. We repeated this process until only statistically significant interaction terms remained. We used bootstrap resampling (k = 5000) with 95% bias-corrected confidence intervals of the indirect effects. For those independent variables with no significant coefficient, we further explored their associations with the duration and frequency of the physical activity using ordinal logistic regression and nonlinear regression.

## 3. Results

### 3.1. Socio-Demographic Factors 

In general, the overweight population was as high as 36% (Chinese standard 24 ≤ BMI < 28) [43]. Only 7.5% of the residents were obese (BMI ≥ 28), lower than the national average [44]. The discrepancy was likely due to the younger age of the online sample. 

Further, one-way ANOVA demonstrated statistically significant differences in health outcomes among individuals, as shown in Table 1. Gender, age, education background, and private car ownership all had significant effects on BMI. Among all participants, 48% were men, who had higher BMI than women and also higher than the normal level (18.5 ≤ BMI < 24). Older respondents were more likely to have higher BMIs, which was in line with previous empirical findings [44]. People with a high school degree or lower education level were overweight. While, gender, age, and private car ownership also had significant effects on physical activity. Additionally, we tested the impact of socio-demographic groups on SoC. We found a bimodal relationship between income and SoC, with both higher- and lower-income respondents having higher SoC, compared to middle-income respondents. 

Additionally, we found physical activity had impacts on self-reported health status, namely that the MPA frequency duration and the total time of walking related to mental health status. Physical activity can somehow benefit the phycological aspect.

Finally, health indicators were significantly correlated with self-reported health status. That is, BMI was associated with hypertension (r = 0.104, *p* < 0.01) and hyperglycemia (r = 0.237, *p* < 0.01), and the SoC had a relationship with mental subhealth (r = −0.094, *p* < 0.05). We also found most of the residents were physically and mentally healthy, but up to 19% chose mental subhealth. This index was much higher than the common chronic diseases, like cardiovascular, cerebrovascular diseases, and hypertension, which showed that residents’ mental health was also worthy of attention.

### 3.2. Objectively Assessed Environment

Table 2 summarizes the results of the regression model examining the impact of the objectively assessed environment and health outcomes. We found four statistically significant independent variables; land use mix, street connectivity, accessibility, and net residential density. Land use mix and road network density (one of the connectivity indicators) was positively related to the duration of VPA. Additionally, the number of public facilities had a positive effect on the total time of VPA, but weak correlation coefficient. Besides, number of public facilities was also positively related to walking time. In contrast, net residential density had a curvilinear correlation with MPA, and the interpretation rate of the model was the highest among all built environment fitting models (R^2^ = 0.628, *p* < 0.001). Further, density and connectivity were inversely associated with the sense of community. Density included building density and FAR, and the impact of building density was relatively weak, compared to FAR. As for BMI, the slope was the only indicator that had a significant negative impact. However, no significant relationships were observed between objective and perceived street-level terrain slopes.

The perceived and objectively measured neighborhood environment were related but distinctly constructed for unique variance in physical activity in the previous studies [45]. Similarly, we found comparably defined variables exhibited low agreement between each other. That is, the number of facilities, road network density, and FAR had associations with the perceived neighborhood environment but not with the conceptual corresponding one. More specifically, the number of catering, leisure facilities, and public transit stops had associations with the social environment. Road network density and FAR were related to aesthetic and perceived social environment. Therefore, in the mediation test, only FAR was mediated by the social environment with sense of community. However, socio-demographic variables did not show any significant difference in mediation models.

### 3.3. Perceived Neighborhood Environment

Socio-demographic attributes showed a significant difference in the perceived neighborhood environment. Men had a higher perceived neighborhood environment score than women, especially in safety and aesthetics. Those with a high education background showed lower social interaction with neighbors. Additionally, perceived neighborhood environment total score had a positive association with the social environment, implying that residents who perceived higher scores on environment quality tended to have a stronger sense of community.

Results of regression analysis about the perceived environment and health outcomes are summarized in Table 3. Only aesthetics and safety had a significant impact on physical activity. Aesthetics was positively related to both frequency and duration in MPA and more frequency in VPA, which was mediated by the social environment. Additionally, safety was related to the total time of VPA. Further, perceived social environment mediated the associations (main effects) of five out of six perceived neighborhood environment with sense of community, namely connectivity, accessibility, aesthetic, sidewalk, and safety. Finally, we found only connectivity among all the perceived neighborhood attributes was mediated by the social environment on BMI. Although income had no direct effect on BMI, it moderated the effect between connectivity and social environment in the mediation effect. 

## 4. Discussion

### 4.1. Direct Effect of Environment

Density and connectivity were positively correlated to physical activity, but the regression coefficient was weak, even statistically significant, which was in line with previous studies [46]. We found road network density had a positive association with the duration of VPA, and the number of intersections was correlated to MPA. This was due to the compact land use that can reduce residents’ daily shopping distance, enhance neighborhood commerce, and thus promote residential walking [4]. In contrast, land use mix and connectivity had inverse impacts on sense of community, implying that these two characters had more complicated associations between physical and mental health. Further, the number of parks within the community was related to VPA as expected, because parks in the neighborhood can facilitate engagement in leisure-time physical activity [47]. In addition to the park, this study added other service facilities that might encourage recreational physical activity [48], including gyms, outdoor sports venues, and fitness places, which also showed positive associations. Rather, recreational sports venues contributed to physical activity and were the only elements in the objectively assessed environment that were significantly related to the total time of physical activity, potentially owing to the extension of the activity choice. 

We additionally took topographic factors into account, especially the hilly terrain. Terrain topography changes in the spatial variability is obvious within the research units. However, slope exhibited no significant associations with physical activity, but negative associations with BMI. That is, respondents had lower BMI in areas with steeper slopes. Further, no mediation or moderation effects were found between slope and BMI, which needs further study on the associations between topography and health outcomes.

The evidence for the association between built environment and physical activities comes mainly from self-reported environmental perceptions [16]. Particularly, we found aesthetics to play an essential role between built environment and physical activity. Factors contributing to aesthetic characteristics in our study included night lighting, street trees, pedestrians, architectural aesthetics, natural scenery, and interesting things within the neighborhood. Furthermore, traffic safety had no significant effect on physical activity, but the impact of security safety was significant. What we found was partly consistent with the literature was that not all aspects of safety have a significant impact on PA, namely that security and traffic safety were sometimes not significant, while, the main effect of pedestrian safety was significant [25]. 

Objectively assessed environment and perceived environment were related, that is, a higher objective walkability score was associated with a higher perceived neighborhood environment score which, in turn, was associated with higher odds of meeting physical activity recommendations [20]. However, our results showed that only land use mix and construction year was significant, albeit inconsistently, correlated with perceived neighborhood environment score. More specifically, industrial frequency density (one of the land use mix indicators) had a negative association while the later had a positive impact, which was consistent with the practical experience; the newly built residential areas had relatively preferable environmental quality, but the adjacent industries would discourage the perceptions of the environment.

### 4.2. Indirect Effect of Environment

Previous studies have found that perceived neighborhood environment mediated the association between built environment and physical activity. In particular, the impact of the number of parks on physical activity was mediated by their perceptual corresponding environment. The effects of intersections and land use mix on physical activity were moderated by gender and safety [19]. Additionally, gender and educational background moderated the effect of perceived safety on physical activity [21]. However, our research found no mediation effect of perceived environment, which was inconsistent with the existing evidence. We found that the number of service facilities was the only objectively assessed environment element significantly related to their perceptually corresponding environment, but there was no mediation effect. Although gender had a significant effect on both perceived safety and physical activity, no interaction effect was found. The possible explanation was that the NEWS scale had a measurement error in a high-density situation.

The research confirmed that the social environment in the high-density living environment had important significance in promoting physical activity and sense of community. Sense of community can promote people’s awareness of safety, comfort, and self-confidence, thereby stimulating positive physical activities in the community and increasing opportunities for social interaction [49]. Previous study found that low density and abundant commercial land were conducive to sense of community, which can promote leisure activities [37]. Likewise, our findings identified a significant positive impact of sense of community on all types of activities. However, high density negatively affected the sense of community, that might be owing the cramped feelings and lack of green space resulted by high density, which were key barriers to facilitate social interactions. Additionally, residents with positive perceptions of their neighborhood characters have a better social network. Well maintained social relationships contribute to the cultivation of sense of community, which is beneficial to both physical and mental health.

### 4.3. Strengths and Limitations

There are several strengths and limitations to our study. Although great achievements have been made in the study of the built environment and physical activity in the past decades, there is not much research on the high-density environment in China [50,51,52]. The built environment we identified significantly associated with physical activity supported previous studies’ findings [9,10,13] and also provided further evidence on Chinese urban environment (Appendix B). We found that both objective (density, connectivity, and accessibility) and subjective (aesthetics and safety) environment were positively related to physical activity which is in line with the literature, but density and connectivity had inverse impacts on sense of community. The study focused on the social environment as the main moderating variable, finding that the social environment had essential significance for physical activity and health outcomes. We confirmed the importance of social environment in the high-density living environment in promoting physical and mental health.

However, there were still limitations. First, the study controlled for individual characteristics but did not provide specific analysis of behavioral differences among population groups. Therefore, it would be useful for future studies to distinguish between different demographic groups in the neighborhood with hilly terrain. Second, we only used the buffer method to define neighborhood boundary and made no distinction between the cognitive distance in the questionnaire and the actual buffer distance, which may result in the mismatch between objective and perceived environment.

Finally, the demography of the respondents obtained through the Internet survey tended to be younger than the local demography, and the neighborhood self-selection bias of the respondents remained unresolved. Additionally, the sample size was limited to an acceptable range of errors due to the difficulty in data collection. However, the study limited the study population within the central districts and focused on those who had convenient access to public social media, such as white-collar adults in urban centers. The sampling design and data cleaning process also alleviated the concerns related to sampling error. In China’s non-first-tier cities, city-level data from authoritative databases is not rich and difficult to obtain publicly, especially those related to personal information and health. For a more comprehensive understanding of the relationship between high-density living environment and health in China, this needs to be further explored. 

## 5. Conclusions

In general, this study demonstrated that both objective and perceived environment factors had significant impacts on physical and mental health in Dalian. The association of density, connectivity, the availability of service facilities, neighborhood quality with physical activity and/or walking, which has been found to be linked in research in first-tier cities, also holds true in Dalian. Especially, we found that aesthetics and safety play an essential role between built environment and physical activity. This research provides new insights that high density facilitated engagement in physical activity but hindered the sense of community which also had influences with physical activity. Additionally, the objectively measured slope was related to BMI; residents living in the neighborhood with more steep slopes had lower BMI, indicating that the design of slope might be influential. Given the complexity of density in Chinese neigborhoods, especially with hilly terrain, we suggest that future work of this nature might aim to identify to what extent density can facilitate both physical and mental health. This study is also part of an evidence base that social environment is of equal importance, compared to built environment, which needs to be well established by local officials or developers in Chinese communities.

## Figures and Tables

**Table 1:** Associations of socio-demographic factors on physical activity and health outcomes.

		Physical Activity (min/week)						Health Outcome	
		VPA Mean (SD)	F/P	MPA Mean (SD)	F/P	Walking Mean (SD)	F/P	BMI Mean (SD)	F/P
Gender									
Male	48%	93 (125)	11.90	116 (133)	2.86	155 (135)	0.037	24.5 (4.3)	28.4
Female	52%	58 (92)	0.001	96 (117)	0.091	152 (120)	0.848	22.1 (4.9)	0.000
Age									
22–32	48%	62 (96)		82 (112)		153 (125)		22.3 (3.6)	
33–43	29%	72 (110)	3.32	103 (121)	5.96	142 (128)	1.13	24.1 (6.1)	4.10
44–54	16%	96 (125)	0.006	156 (142)	0.000	163 (129)	0.346	24.3 (5.4)	0.001
55–65	7%	112 (108)		163 (129)		166 (123)		25.1 (2.8)	
Income									
Low	17%	68 (115)		98 (140)		148 (137)		23.3 (5.9)	
Medium-low	27%	70 (106)		96 (124)		162 (131)		22.4 (3.9)	
Medium	21%	59 (92)	1.50	96 (114)	1.20	158 (132)	0.133	23.7 (5.1)	1.20
Medium-high	11%	105 (124)	0.20	117 (118)	0.313	154 (134)	0.97	23.4 (5.7)	0.31
High	24%	79 (115)		128 (134)		155 (115)		23.7 (3.8)	
Education									
Middle school	2%	48 (118)		66 (156)		96 (94)		25.4 (9.6)	
High school	6%	90 (141)		80 (105)		122 (132)		26.6 (9.2)	
Junior school	9%	71 (111)	0.77	125 (137)	0.69	157 (131)	1.67	23.7 (4.1)	4.46
University	45%	81 (120)	0.54	109 (131)	0.59	163 (133)	0.16	22.2 (4.4)	0.002
Master or higher	38%	63 (89)		104 (122)		158 (122)		23.2 (3.8)	
Private car									
None	40%	63 (102)	3.52	93 (122)	3.88	170 (132)	1.25	22.7 (4.8)	4.16
One or more	60%	80 (114)	0.061	116 (130)	0.050	148 (126)	0.26	23.6 (4.7)	0.042

VPA: Vigorous physical activity, MPA: Moderate physical activity, BMI: Body mass index, F/P: F value and P value.

**Table 2:** Associations of objectively assessed built environment on physical activity and health outcomes.

Built Environment	VPA (min/week)		MPA (min/week)	Walking (min/week)		SoC		BMI	
	Coefficient	95% CI		Coefficient	95% CI	Coefficient	95% CI	Coefficient	95% CI
Land use mix									
Commercial frequency ratio	0.3602 ** (0.0755, 0.6448) (Duration)								
Density									
Residential density			(Duration)			−0.098 ** (−0.1777, −0.0167)			
FAR						−0.1438 *** (−0.2238, −0.0637)			
						b_1_ = −0.0718 ** (−0.1311, −0.0125)			
						b_2_ = 0.6765 **** (0.6166, 0.7364)			
						a = −0.1064 **			
Connectivity									
Road network density	0.4105 ** (0.1241, 0.6970) (Duration)					−0.1190 ** (−0.1996, −0.0348)			
						b_1_ = −0.1159 ** (−0.1948, −0.0370)			
						b_2_ = 0.2134 **** (0.1346, 0.2923)			
						M_2_ sobel test *p* = 0.7692			
Intersections				0.134 ** (0.158, 0.947)					
Accessibility									
Facility counts	0.0595 ** (0.0076, 0.1115)			0.1096 ** (0.0288, 0.1903)					
	b_1_ = 0.0609 ** (0.0180, 0.1037)			b_1_ = 0.1169 ** (0.0367, 0.1971)					
	b_2_ = 0.0781 *** (0.0399, 0.1224)			b_2_ = 0.1338 *** (0.0536, 0.2141)					
	M_2_ sobel test *p* = 0.7849			M_2_ sobel test *p* = 0.3019					
Topography									
Slope								−0.5877 ** (−1.0468, −0.1285)	
Construction									
Housing price				0.101 ** (0.638, 25.505)					

* *p* < 0.1, ** *p* < 0.05, *** *p* < 0.01, **** *p* < 0.001. Dependent variables: PA or (duration/frequency), SoC, BMI. M_1_ = perceived environment; M (Mod)_2_ = social environment; Co 3 = socio-demographic gender, age, income).

**Table 3:** Associations of perceived built environment on PA and health outcomes.

Built Environment	VPA (min/week)		MPA (min/week)		Walking (min/week)		SoC		BMI	
	Coefficient	95% CI	Coefficient	95 % CI	Coefficient	95 % CI	Coefficient	95% CI	Coefficient	95% CI
Connectivity							0.199 **** (0.1190, 0.2782)		0.6796 *** (0.2956, 1.0637)	
							b_2_ = 0.6832 **** (0.6227, 0.7437)		sobel test *p* = 0.6816	
							a = 0.2414 **** (0.1626, 0.3202)		b_8_ = 0.0044 ** (0.2923, 1.0829)	
							b_15_ = −0.0722 **		b_10_ = 0.4184 ** (0.0907, 0.7460)	
							M_1_ and Mod_2_ Mediated moderation		Mod_2_ Moderation	
Accessibility							0.2602 **** (0.1818, 0.3387)			
							b_1_ = 0.0727 ** (0.0118, 0.1336)			
							b_2_ = 0.6710 **** (0.6101, 0.7319)			
							a = 0.2795 **** (0.2015, 0.3575)			
							M_1_ Partial mediation			
Slope										
Aesthetics	0.1471 ** (0.0271, 0.2672) (Frequency) b_2_ = 0.2685 ** (0.0389, 0.4981) a = 0.8542 **** (0.8038, 0.9045) M_1_ Mediation		0.1372 ** (0.003, 0.2740) (Frequency) 0.3860 ** (0.1363, 0.6356) (Duration)				0.6261 **** (0.5628, 0.6894) b_2_ = 0.5788 **** (0.4663, 0.6912) a = 0.8543 **** (0.8119, 0.8963) M_1_ Mediation			
Sidewalk							0.1024 ** (0.0216, 0.1832) b_2_ = 0.6911 **** (0.6317, 0.7505) a = −0.1464 ** (0.8119, 0.8963)			
Safety	0.121 ** (3.836, 33.002) b_2_ = 12.73 ** (1.1667, 24.292) a = 0.4128 **** (0.3246, 0.5010) M_1_ Mediation						0.370 **** (0.280, 0.460) M_1_ Mediation b_1_ = 0.1015 ** (0.0252, 0.1778) b_2_ = 0.6494 **** (0.5731, 0.7257) a = 0.4128 **** (0.3246, 0.5010) M_1_ Partial mediation			
Social environment	0.107 ** (1.203, 22.39)		0.097 ** (0.051, 24.59)		0.141 *** (5.854, 22.57)		0.539 **** (0.282, 0.383)		0.131 *** (0.169, 1.086)	

* *p* < 0.1, ** *p* < 0.05, *** *p* < 0.01, **** *p* < 0.001; Dependent variables: PA (duration/frequency), SoC, BMI. M (Mod)_1_ = social environment; Mod_2_ = socio-demographic (gender, age, income).

## Appendix A

**Table A1:** Rotated component matrix.

Questions—Abbreviated	Component					Communalities
	Factor 1	Factor 2	Factor 3	Factor 4	Factor 5	
Stores are within easy walking distance	0.066	**0.704**	0.021	0.171	−0.031	0.531
Many places to go within easy walking distance	0.15	**0.774**	−0.111	0.046	0.046	0.638
Easy to walk to a transit stop	0.043	**0.694**	0.128	0.17	−0.251	0.592
Distance between intersections is usually short	−0.03	**0.725**	0.081	0.145	0.047	0.556
Many alternative routes for getting from place to place	0.214	**0.708**	−0.045	−0.115	0.043	0.564
The streets in my neighborhood are hilly, making it difficult to walk in	−0.081	−0.118	0.256	0.315	**0.674**	0.64
Major barriers make it hard to walking from place to place	0.16	0.069	0.347	−0.156	**0.717**	0.69
Sidewalks are separated from the road/traffic by parked cars	−0.1	−0.01	**0.823**	0.042	0.038	0.691
Grass/dirt strip separates the streets from the sidewalks	−0.018	0.034	**0.813**	−0.09	0.212	0.716
My neighborhood streets are well lit at night	**0.625**	0.205	−0.079	0.085	−0.102	0.456
Walkers on the streets in my neighborhood can be easily seen by people	**0.446**	0.196	0.24	0.449	−0.358	0.625
There are trees along the streets in my neighborhood	**0.683**	0.111	−0.056	0.296	−0.138	0.589
There are many interesting things to look at while walking	**0.774**	0.133	0.027	0.048	−0.007	0.619
There are attractive buildings/homes in my neighborhood	**0.838**	0.036	−0.148	0.074	0.167	0.758
There are many attractive natural sights in my neighborhood	**0.779**	−0.059	−0.193	0.138	0.17	0.695
There is so much traffic along nearby streets that it makes it difficult to walk	0.163	−0.023	−0.633	0.043	−0.119	0.444
The speed of traffic on most nearby streets is usually slow	0.189	0.178	−0.212	**0.635**	0.267	0.587
The crime rate in my neighborhood makes it unsafe	0.263	0.162	−0.007	**0.697**	−0.062	0.585
KMO value	0.801					−
Bartlett test of spherical	2287.768					−
df	153					−
*p* value	0					−

Extraction method: Principal Component Analysis.

## Appendix B

**Table A2:** Comparison between the results of the research and the values at national level.

Level	Built Environment Elements Associated with Physical Activity and Health Outcomes			
	Direct Effect		Indirect Effect	
	Positive	Negative	Mediator	Main Effect
This study	(Physical activity) land use mix, connectivity, accessibility, density, aesthetic and safety neighborhood quality, aesthetics and safety	(SoC) density connectivity (BMI) slope	Social environment	Aesthetic and safety on physical activity, perceived connectivity, accessibility, aesthetic, sidewalk, and safety on SoC
Other Chinese cities	Land use mix [9], accessibility [10], neighborhood quality [6], design features and safety [53]	(Physical activity) density, ingle function, adjacent main road [9]		
Western countries	Land use mix, density, connectivity, accessibility pedestrian infrastructure, parks/squares [12,13,14,15], coasts/hills/sceneries, well-maintained neighborhoods, aesthetics and safety [54]	(Physical activity) city sprawl, unpleasant vistas, ill-maintained roads and facilities, dirty environment, garbage, broken glass [54].	Perceived number of parks	Parks on physical activity [19]
			Gender and safety	Intersections and land use mix on physical activity [19]
			Gender and education	Safety on physical activity [21]
